# Evaluating Muscle Damage Biomarkers in Adolescent Athletes: Implications for Public Health in Tacna, Peru—2023

**DOI:** 10.3390/ijerph21111394

**Published:** 2024-10-23

**Authors:** Kelly Geraldine Yparraguirre Salcedo, Anthony Brayan Rivera Prado, Luis Lloja Lozano, Vicente Freddy Chambilla Quispe, Claudio Willbert Ramirez Atencio

**Affiliations:** 1 Laboratory of Regenerative Medicine, Universidad Nacional Jorge Basadre Grohmann, Avenida Miraflores S/N, Ciudad Universitaria, Tacna 23003, Peru; ariverap@unjbg.edu.pe (A.B.R.P.); lllojal@unjbg.edu.pe (L.L.L.); vchambillaq@unjbg.edu.pe (V.F.C.Q.); cramireza@unjbg.edu.pe (C.W.R.A.); 2 School of Medicine, Universidad Nacional Jorge Basadre Grohmann, Avenida Miraflores S/N, Ciudad Universitaria, Tacna 23003, Peru

**Keywords:** muscle damage, serum values, athletes, biochemical markers

## Abstract

The success of an athlete is influenced by various factors, including nutritional status, physical condition, training level, recovery, psychological factors, and genetics. Studies on athletes often take a preventive approach to evaluate muscle fatigue caused by intense physical exercise, which can impair performance, particularly in soccer players. This study aims to determine the serum levels of biochemical markers of muscle damage in adolescents from sports centers in the city of Tacna. A sample of 326 adolescents who met the inclusion and exclusion criteria was analyzed for muscle damage markers, including CRP, LDH, CK-total, and CK-MB, to assess potential skeletal muscle injury. The results were as follows: for the LDH test, 2.5% had low-level values, 91.4% had values within the normal range, and 6.1% had high values; for the CK-total test, 74.8% were within normal limits, and 25.2% had elevated values; for the CK-MB test, 99.1% had normal values, and 0.9% had elevated values; and for the CRP test, 95.7% were negative, and 4.3% were positive. According to the CK-MB/CK relative index, 7.1% of adolescents were suspected of having muscle damage. This study concludes that there are alterations in the biochemical markers of muscle damage in adolescents from the sports centers in Tacna.

## 1. Introduction

Muscle damage is a common issue among athletes due to the significant mechanical and metabolic workload involved in physical activity. When this activity is performed continuously and competitively with macro and microcycles of training, a series of adaptations occur that maximize muscle performance. However, excessive training without adequate recovery can lead to chronic fatigue syndrome, which may severely impact athletic performance. Therefore, biochemical markers of muscle damage are used to prevent and diagnose muscle injuries. These biomarkers are used to diagnose the injuries and not to directly prevent them [1].

Currently, the most common marker for diagnosing muscle damage is Creatine Kinase (CK). Although the timing of its peak value post-exercise is imprecise, ranging from 24 to 72 h, CK is a cytosolic marker predominantly found in skeletal muscles and the heart and in smaller amounts in the brain. There are three types of CK enzymes: CK-MM, which is frequently found in skeletal muscles; CK-MB, which is present in the myocardium; and CK-BB, which is found in the brain [2,3]. 

Another commonly used enzyme for muscle damage assessment is Lactate Dehydrogenase (LDH). This enzyme acts on pyruvate and lactate through the interconversion of adenine-nicotinamide dinucleotide (AND). LDH has five isoenzymes present in living cells, which are formed by the combination of M and H polypeptides. Elevated levels of LDH can reflect phenomena such as hemolysis, osteoblastic activity, neoplastic proliferation, cellular damage, and necrosis [3,4].

C-Reactive Protein (CRP) is another enzyme considered an indicator of inflammation, which can also signal muscle damage. Elevated CRP levels are associated with infections, trauma, surgery, and chronic inflammatory conditions. In the context of intense physical activity, high CRP levels can occur. However, continuous training may lead to an adaptation where baseline levels are regulated [3].

Sports professionals and athletes need to be aware of their muscular condition to anticipate and identify the level of muscle injuries and subsequently apply appropriate treatment and establish training breaks and rehabilitation. Therefore, the aim of this study is to determine the level of muscle damage according to biochemical markers and serum values in adolescents from the sports centers in Tacna.

## 2. Materials and Methods

### 2.1. Design and Study Population

This study is descriptive and cross-sectional, with blood samples collected at a single time point. The participants are from various soccer sports programs in the city of Tacna, aged 13 to 18 years. The first phase was conducted at Videna Tacna (Tacna Departmental Football League), and the second phase took place at the biology laboratory in the Faculty of Sciences for sample processing and biochemical analysis.

The population consisted of all adolescents from various sports programs in the city of Tacna who participated in the health campaign. All participants underwent a medical examination and laboratory tests. The sample included 376 adolescents who met the inclusion and exclusion criteria.

### 2.2. Criteria for Inclusion

- Healthy athletes aged between 13 and 18 years.
- All soccer players who were actively training during the three months prior to sample collection.
- Individuals who have undergone a complete medical examination.
- Participants who signed the informed consent form.
- Participants who complied with the fasting requirements.

### 2.3. Criteria for Exclusion

- Participants with any chronic disease.
- Participants who have been inactive for less than three months.
- Participants currently undergoing treatment with the following medications: Amphotericin B, fibrates, statins, steroids such as dexamethasone (for CK), and aspirin, clofibrate, fluorides, mitramycin, and procainamide (for LDH).
- Participants exhibiting any alteration in clinical inflammation parameters due to other causes (e.g., Erythrocyte Sedimentation Rate (ESR)).
- Participants who have not signed the informed consent form.

### 2.4. Selection of Biomarkers for Muscle Damage Assessment

The biomarkers selected for this study (CRP, LDH, total CK, and CK-MB) were chosen because they are the most commonly used for detecting skeletal muscle damage in previous studies involving athletic populations. These markers are well-accepted in the scientific literature due to their ability to quickly detect muscle damage in response to intense physical activity. Although other biomarkers, such as myoglobin, AST, troponin, and aldolase, are useful, they are more commonly employed in cardiac or specific muscle disease contexts, which was beyond the scope of this study. Our focus was on biomarkers related to skeletal muscle damage that reflect the impact of athletic activity in adolescents.

### 2.5. Sample Collection

Venous blood collection was carried out following the protocols established by the Ministry of Health [5], ensuring sterile conditions and proper sample handling to avoid hemolysis and other interferences. Participants were registered before entering the sample collection room.

A puncture site was selected, typically from veins in the elbow crease: the basilic, cephalic, or median cubital veins. A tourniquet was applied 5 to 10 cm above the puncture site using a slipknot. The individual was instructed to clench and unclench their fist to make the vein more prominent; then, they were asked to relax their hand.

The selected area was cleaned with a cotton swab moistened with 70% ethanol and allowed to dry for a few seconds. The needle adapter was then taken, and the needle’s end to be inserted into the vein was uncovered. Venipuncture was performed with the bevel of the needle facing upward at a 15° angle between the needle and the skin. Blood was collected by inserting the other end of the needle into the tube, penetrating the stopper without touching the disinfected area.

Once the tube began to fill, the tourniquet was removed, and the individual was asked to open their hand. The tube (BD Vacutainer™ Blood Collection Tube with Gel Separator) was allowed to fill to the appropriate vacuum level to minimize the risk of hemolysis.

Finally, the tube with the activator gel was removed and replaced with a tube containing an anticoagulant (BD Vacutainer™ Tube with EDTA). The new tube was filled to the indicated level, after which the needle and adapter were removed. A clean cotton swab was applied with gentle pressure to the puncture site.

### 2.6. Sample Transport

The samples were transported from the Videna to the laboratory in a biological specimen cooler, maintaining an approximate temperature of 2 °C to 8 °C for preservation.

### 2.7. Sample Processing

The samples were removed from the cooler and placed in racks for sorting according to the designated codes to ensure all were complete.

The samples were centrifuged at 4000 RPM for 10 min using a centrifuge (Eppendorf 5804 R) to obtain serum. They were placed in the centrifuge in groups of eight, ensuring that the volume of the tubes (BD Vacutainer™ Blood Collection Tubes) was balanced opposite each other to maintain equilibrium during centrifugation and to prevent accidents.

After centrifugation, the samples were carefully removed from the centrifuge and arranged in racks for counting the number of patients. Plasma was separated from the samples into coded Eppendorf tubes (Eppendorf^®^ Safe-Lock Tubes) according to their respective codes to discard hemolyzed samples and prepare for subsequent tests.

Using a calibrated micropipette set to 100 µL, we carefully transferred plasma to the corresponding Eppendorf tubes according to each patient’s code. The plasma samples in the Eppendorf tubes were then refrigerated at temperatures below 5 °C using a refrigerator for further analysis.

### 2.8. Biochemical Analysis

#### 2.8.1. Total CK

Total CK (creatine kinase) levels were analyzed using the Peak Instruments UV-Vis Spectrophotometer following the manufacturer’s instructions. Serum samples were prepared by centrifugation at 4000 RPM for 10 min using the Eppendorf 5804 R Centrifuge. Total CK activity was measured using the Roche Creatine Kinase Total (CK) Reagent Kit, which includes a substrate solution and a reagent buffer. The assay was performed according to the kit protocol: 100 µL of serum was mixed with 200 µL of the reagent solution in a cuvette. The reaction was incubated at 37 °C and monitored spectrophotometrically at 340 nm using the Peak Instruments UV-Vis Spectrophotometer. The enzymatic activity was quantified based on the change in absorbance, with results reported in U/L.

#### 2.8.2. CK-MB

CK-MB (creatine kinase-MB) levels were quantified using the Peak Instruments UV-Vis Spectrophotometer following the manufacturer’s instructions. Serum samples were first prepared by centrifuging at 4000 RPM for 10 min with the Eppendorf 5804 R Centrifuge to separate the serum from cellular components. CK-MB activity was assessed using the Abcam CK-MB Enzyme Activity Assay Kit, which contains a specific substrate and reagent buffer designed to selectively measure CK-MB activity. For the assay, 100 µL of serum was mixed with 200 µL of the assay buffer in a microplate well. The reaction mixture was incubated at 37 °C for 30 min to allow for enzyme-substrate interaction. Following incubation, the absorbance of the reaction at 340 nm was measured using the Peak Instruments UV-Vis Spectrophotometer. The enzymatic activity of CK-MB was calculated based on the change in absorbance over time, with results reported in U/L.

#### 2.8.3. LDH

Lactate dehydrogenase (LDH) levels were assessed using the Peak Instruments UV-Vis Spectrophotometer as outlined by the manufacturer’s guidelines. Initially, serum samples were obtained by centrifugation at 4000 RPM for 10 min with the Eppendorf 5804 R Centrifuge to separate the serum from cellular debris. The analysis employed the BioVision LDH Activity Assay Kit, which provides a specific substrate and reagent buffer for accurate measurement of LDH activity. In this assay, 50 µL of serum was combined with 200 µL of the provided assay buffer in a microplate well. Following this, 50 µL of the LDH substrate solution was added to initiate the reaction, which was then incubated at 37 °C for 30 min. The change in absorbance was recorded at 450 nm using the Peak Instruments UV-Vis Spectrophotometer. The LDH activity was determined based on the absorbance changes, with results expressed in units per liter (U/L).

#### 2.8.4. CRP

To measure C-reactive protein (CRP) levels, we used the Peak Instruments UV-Vis Spectrophotometer following established protocols. Serum samples were first processed by centrifugation at 4000 RPM for 10 min using the Eppendorf 5804 R Centrifuge to obtain clear serum. The CRP levels were quantified using the Abcam CRP Enzyme-Linked Immunosorbent Assay (ELISA) Kit, which included a specific anti-CRP antibody and reagent solutions. For the assay, 100 µL of serum was added to each well of a microplate pre-coated with CRP-specific antibodies. After adding 100 µL of the enzyme-conjugated secondary antibody and incubating at 37 °C for 1 h, the wells were washed, and 100 µL of substrate solution was added. The reaction was stopped after 15 min, and absorbance was measured at 450 nm using the Peak Instruments UV-Vis Spectrophotometer. CRP levels were determined by comparing the absorbance to a standard curve, with results reported in mg/L.

#### 2.8.5. CK-MB/CK

The relative index of CK-MB/CK was determined using a formula-based approach. Initially, serum samples were processed by centrifugation at 4000 RPM for 10 min with the Eppendorf 5804 R Centrifuge to isolate the serum. The levels of CK-MB and total CK were measured using the Peak Instruments UV-Vis Spectrophotometer in conjunction with commercial assay kits: BioVision CK-MB Enzyme-Linked Immunosorbent Assay (ELISA) Kit for CK-MB and BioVision CK Total Assay Kit for total CK. Following the manufacturer’s instructions, the absorbance values for both CK-MB and CK were recorded at 450 nm. The CK-MB/CK ratio was calculated using the formula:

$$Relative\;Index\;CK-MB/CK=\;\frac{CK-MB\;concentration\;(U/L)}{Total\;CK\;concentration\;(U/L)}$$

This ratio was used to assess the proportion of CK-MB relative to total CK, providing insights into potential myocardial damage. Based on the relative index, the following classifications were applied: a CK-MB/CK ratio of less than 5% typically indicates no significant myocardial injury; a ratio between 5% and 10% suggests mild myocardial damage; and a ratio exceeding 10% is indicative of significant myocardial injury.

### 2.9. Statistical Analysis

Data were managed using Microsoft Excel 2016 spreadsheets, which contained information on clinical results and laboratory test outcomes for the study participants. Informed consent was obtained from all participants prior to data collection. Initially, the collected data were organized in Microsoft Excel 2016 to establish a database of all patients who met the inclusion criteria. The information was then prepared for further analysis using IBM SPSS Statistics 25. Descriptive statistics were applied to determine frequencies and create distribution graphs for the variables. This analysis aimed to assess whether the biochemical test results were altered and to categorize serum values as high, normal, or low, thereby evaluating muscle damage.

### 2.10. Ethical Statement

The research project was submitted to the Research Ethics Committee of the Hipolito Unanue Regional Hospital in Tacna, Peru, and approved under protocol No. 010-CIEI-2023, issued on 4 May 2023.

## 3. Results

Table 1 presents the statistical analysis for each biochemical marker, including the mean, median, mode, standard deviation, variance, minimum, and maximum values based on the serum levels observed in each patient.

The results from the LDH test were obtained for all adolescents who met the inclusion and exclusion criteria. After discarding unsuitable samples, data analysis was conducted by applying frequency distributions according to serum value scales: low (less than 200 U/L), normal (200–400 U/L), and high (greater than 400 U/L). The results are presented in Table 2.

Table 2 shows that according to the serum value scale, 2.5% of the participants have low values, 91.4% have normal values, and 6.1% have high values for the LDH test. The distribution is variable; however, the median value of 360 U/L suggests that the data are skewed toward the higher limit. For the low serum value range, the median is 209 U/L, which is close to the normal limit, with a minimum value of 125 U/L. In the case of the high-value range, the median is 435 U/L, which is near the normal limit, and the maximum value is 545 U/L.

The results for the total CK test were obtained from all adolescents who met the inclusion and exclusion criteria. After discarding unsuitable samples, data analysis was performed, categorizing the serum values into low (<24 U/L), normal (24–195 U/L), and high (>195 U/L) ranges. The results are presented in Table 3. It is observed that no adolescents had low serum values. Of the total, 74.8% had normal values, while 25.2% had elevated values for the total CK test.

As indicated in Table 4, the CK-MB levels were assessed in all adolescents who met the inclusion and exclusion criteria. After excluding invalid samples, data were analyzed by applying frequency distributions based on the serum value scale: normal (0–25 U/L) and elevated (greater than 25 U/L). As shown in Table 5, 99.1% of the adolescents had normal serum levels, while 0.9% exhibited elevated CK-MB levels.

The results obtained for CK-total and CK-MB were used to calculate the CK-MB/CK ratio. According to the literature, this ratio is crucial for accurate laboratory interpretation and clinical management. A CK-MB/CK ratio of less than 4% suggests a potential muscle injury; a ratio between 4% and 25% indicates possible ischemic cardiopathy; and a ratio greater than 25% points to the possibility of Macro-CK (type I or II) or CK-BB, although ischemic cardiopathy cannot be ruled out and further testing for troponin I may be necessary. The results based on these criteria are summarized in Table 5.

According to the data observed in Table 6, the results reveal that 7.1% of the adolescents exhibit a CK-MB/CK ratio below 4%, suggesting a potential muscle injury. Meanwhile, 92.9% of the adolescents fall within the range of 4% to 25%, indicating either normal conditions or a possible ischemic cardiopathy. No cases were observed with a CK-MB/CK ratio exceeding 25%.

Regarding the PCR test, this assay employs a suspension of polystyrene particles sensitized with anti-human PCR antibodies. When the reagent is exposed to the serum, an antigen-antibody reaction occurs, evidenced by the agglutination of latex particles, which form easily visible aggregates. This allowed us to determine whether the reaction was positive or negative. Following the laboratory analysis, data analysis was conducted to determine the frequencies, and the results are presented in Table 6.

In Table 6, for the determination of CRP, it is observed that 95.7% of the adolescents have a negative result for the agglutination test, while 4.3% have a positive result.

Regarding the number of altered markers, it was observed that out of the 326 adolescents representing the total sample, 35 exhibited alterations in their serum values. As shown in Table 7, the distribution of these alterations is categorized according to the number of altered markers. Table 7 details how many adolescents exhibited alterations in one marker, two markers, three markers, and all four markers used in this study.

According to the table, we can observe that the total number of adolescents with altered serum values, meaning values outside the normal range, is 122 adolescent athletes. Of these, 88.4% show an alteration in only one marker, 9.8% show alterations in two markers, 1.8% of adolescent athletes show alterations in three markers, and no athletes present alterations in all four markers.

Table 8 shows the levels of key muscle damage markers in individuals with elevated values. CK levels range from 190 to 270 U/L, and LDH levels vary between 390 and 450 U/L, indicating significant muscle damage. CRP values are between 1.1 and 1.8 mg/L, suggesting mild to moderate inflammation associated with muscle damage. CK-MB levels, ranging from 19 to 24 U/L, exhibit moderate variability, which may reflect individual differences in muscle impact. These results highlight the variability in the extent of muscle damage and inflammation among the studied individuals, providing a comprehensive view of the biomarkers involved in muscle damage processes.

Table 9 presents the correlation matrix among muscle damage markers. The correlation between CK and LDH is moderate (r = 0.260), suggesting a weak relationship between these two markers. CK shows a weak positive correlation with CRP (r = 0.237), indicating a minor association between muscle damage and inflammation. Conversely, CK has a weak negative correlation with CK-MB (r = −0.143), which suggests a slight inverse relationship. LDH and CRP exhibit a moderate positive correlation (r = 0.483), indicating a more pronounced association between muscle damage and inflammatory response. Notably, LDH and CK-MB have a strong positive correlation (r = 0.780), suggesting that higher LDH levels are associated with increased CK-MB levels. These correlations provide insight into the interrelationships among the biomarkers, highlighting varying degrees of association and potential overlaps in their roles in muscle damage and inflammation.

## 4. Discussion

Related studies on athletes agree on the importance of conducting preventive analyses of sports activities. This helps to identify early alterations in the serum values of muscle damage markers, which could serve to predict injuries that may limit physical performance. During adolescence, many metabolic changes occur in the human body. In athletes, the activities performed demand an accelerated metabolism, especially the need for nutrients and the intracellular processes triggered by increased physical activity. Therefore, it is important to monitor muscle health through biological markers of muscle damage.

LDH (Lactate Dehydrogenase) is an enzyme found in many tissues of the body, with higher concentrations in the heart, liver, kidneys, muscles, red blood cells, brain, and lungs. It is involved in anaerobic energy metabolism, reducing pyruvate from glycolysis to lactate. When muscle damage occurs, meaning the destruction of muscle fibers, serum LDH levels increase significantly. LDH also has a variety of isoenzymes specific to different tissues, providing additional information about the source of muscle damage (Palacios, 2015). In the LDH test, it was found that 2.5% of subjects had low serum levels, 91.4% had normal serum levels, and 6.1% had high serum levels. These results are similar to those found by Macero in 2021, where 98.2% of subjects had normal values, 1.4% had high values, and 0.4% had low values, indicating that the majority of adolescents have normal values. However, LDH behaved similarly in other sports disciplines [6].

According to Aranda’s “Interpretation of Lactate Dehydrogenase” (2010), normal serum parameters depend on the patient’s age. In adults, the range is 50–150 U/L; in children under one year of age, it is 170–580 U/L; in children aged one to nine years, it is 150–500 U/L; and in children aged 10 to 19 years, the range is 120–330 U/L. This is consistent with the results found in our study, where most of the adolescents have values between 200 and 400 U/L, in accordance with the age of the population studied [4].

In our study, the determination of CK-total (Creatine Kinase) found that 0.00% had low serum levels, 74.8% had normal serum levels, and 25.2% had high serum levels. This is consistent with the findings of Macero in 2021, who found that 95.5% of adolescents aged 14 to 18 years had normal values, 4.5% had high values, and 0% had low values. CK is considered the primary marker of muscle damage because it catalyzes the reversible formation of phosphocreatine from creatine and adenosine triphosphate (ATP), which should be reflected in a significant elevation of normal values [6]. 

CK increases during physical exercise due to the rupture of striated muscle fibers. Its elevation is proportional to the intensity and duration of the exercise, but there is an adaptation in training that causes it to rise less in trained individuals than in sedentary ones. Elevated values compared to the baseline (300 U/L) indicate trauma or overtraining, and its concentration in the blood is useful for monitoring the recovery of normal activity in athletes who may have suffered a previous muscle injury [3]. In our results, although most adolescents fall within the normal range, there is a small percentage with high levels. This may be due to different stages of training, the adjustment of physical load, and the influence of muscle metabolism, which affects the serum concentrations of total CK. This is consistent with Romero’s 2022 findings in a soccer team, where he evaluated pre-game and post-game CK-total values. The results showed that pre-game, the mean was 329.6 U/L for a lost game, 328.9 U/L for a drawn game, and 347.5 U/L for a won game. The post-game mean was 632.5 U/L for a lost game, 702.2 U/L for a drawn game, and 672.5 U/L for a won game. In all cases, the serum values exceeded the normal levels for this test [7].

Marqués (2016) also mentions that high CK-III levels can be affected by training status, and if levels persist at rest, it may indicate overtraining syndrome or inadequate nutritional intake. It is said that CK-III serum activity peaks between 6 and 24 h post-exercise and returns to normal within 48–72 h. In soccer players, concentrations typically range between 83 and 1492 U/L [8]. 

In the determination of CK-MB, it was found that 0% had low serum levels, 99.1% had normal serum levels, and 0.9% had high serum levels, similar to what Macedo found in 2021, where 97.7% had normal values, 2.3% had high values, and 0% had low values. These results indicate that the majority of adolescents show normal values, consistent with our findings [6]. 

The relationship between CK-MB and CK-total, also known as the relative index, can help in the interpretation of laboratory results and thus improve patient diagnosis. In our results, we found that 7.1% of adolescents had an index lower than 4%, 92.9% had an index greater than 4% but less than 25%, and no participant had an index greater than 25%. Some authors suggest that when the index is >25%, it may indicate a myocardial rather than skeletal elevation of CK-MB, but this would need to be confirmed with a Troponin I determination. When the index is <4%, it means that the total CK value has increased or the CK-MB value is low, suggesting muscle injury. This is explained by the fact that the total CK percentage includes the CK-MM values, which are specific to the myofibrillar M-line structure located in the sarcomere, where 5–10% of the total CK-MM is found [7]. 

The CK-MB/CK index suggests the presence of muscle damage in 7.1% of adolescents. While these findings are indicative, we recommend additional studies that include troponin I measurement, which is more specific to rule out potential cardiac injuries and ensure the accuracy of the interpretation of the observed muscle damage biomarkers in athletes.

For the CRP (C-reactive protein) marker in this study, it was found that 95.7% of adolescents had a negative result, and only 4.3% had a positive result. This is because prolonged or inappropriate physical activity during sports training often generates an inflammatory process. CRP is a protein induced by IL-6 in the liver, and it is evidenced by sensitive biomarkers of inflammation, infection, and tissue damage in response to exertion [9,10].

It has been observed that the majority of patients in the CRP results do not present an alteration, which is due to the fact that CRP levels increase rapidly during a high-level inflammatory process and decrease rapidly with excretion between 4 and 9 h [6]. 

Another advantage is that CRP is not affected by conditions such as anemia, polycythemia, protein levels, red blood cell shape, sex, or an important factor like the patient’s age and other types of inflammatory diseases. For this reason, the sedimentation rate was applied within the exclusion criteria, as this test is affected by all these traits [11].

In our view, C-reactive protein could be used as a biomarker for tissue trauma and injury; this is consistent with our study because the positive values found for tissue inflammation coincide with the values found for total CK [12].

The data obtained from this study, particularly those related to elevated CK levels and other muscle damage biomarkers, have significant implications for the early detection of subclinical neuromuscular and musculoskeletal diseases. Previous research has shown that late-onset muscular dystrophies, such as limb-girdle muscular dystrophy (LGMD) type 2B/R2, are associated with mutations in the DYSF gene and that persistent alterations in serum CK levels can serve as early markers of these degenerative muscle processes [13,14].

Furthermore, longitudinal monitoring of CK levels, along with other markers such as LDH and CK-MB, could facilitate the identification of at-risk individuals, allowing for the implementation of confirmatory genetic studies or more specific muscle assessments such as electromyography or muscle biopsy [15,16]. This preventive approach is crucial for the early diagnosis of neuromuscular disorders, many of which, such as facioscapulohumeral muscular dystrophy and Miyoshi distal myopathy, are slow-progressing and may not present clinical signs until advanced stages [17,18]. Therefore, the results suggest the importance of thorough follow-up of individuals with persistently elevated CK levels, even in young and seemingly healthy populations, such as those evaluated in this study.

A significant limitation of this study is the absence of a control group composed of adolescents not exposed to intense physical activity. Including such a group would have allowed for a direct comparison of biomarker levels and provided a clearer baseline to assess muscle damage. Future research should incorporate a control group to evaluate the differences between athletic and non-athletic adolescents, enabling a more precise interpretation of muscle damage biomarkers in sports contexts.

Additionally, this cross-sectional study provides valuable insights into the biomarker levels of muscle damage at a single point in time. However, a longitudinal design would allow for a more detailed analysis of how these biomarkers fluctuate in response to variations in training load and recovery periods. Future longitudinal studies could improve our understanding of the processes of muscle adaptation and recovery in adolescent athletes.

The results obtained in this study are specific to the adolescent athletes from Tacna, Peru, and may not be fully applicable to other regions or countries due to differences in environmental factors, geographic conditions, and training regimes. We recommend that future studies include populations from various geographic and environmental contexts to verify the generalizability of these findings and provide a broader understanding of the impact of athletic activity on muscle damage biomarkers.

## 5. Conclusions

There are alterations in the biochemical markers of muscle damage in adolescent athletes from sports academies in the city of Tacna. A sample of 326 adolescent athletes was obtained. They complied with the sampling process and met the inclusion and exclusion criteria. Regarding the LDH test, it was found that 2.5% of adolescents had low levels, 91.4% had normal levels, and 6.1% had high levels. For the CK-total test, 74.8% were within the normal range and 25.2% had high levels. The CK-MB test revealed that 99.1% were within normal values, with only 0.9% presenting high levels. The CRP test showed that 95.7% of the adolescents had a negative result, while 4.3% had a positive result. Additionally, according to the relative CK-MB/CK index, 7.1% of the adolescent athletes show evidence of muscle damage.

## Figures and Tables

**Table 1:** Descriptive Statistical Analysis of Biochemical Markers for Muscle Damage.

	LDH	TOTAL-CK	CK-MB	CRP
Number of athletes	326	326	326	326
Mean	327.95	165.76	11.71	1.04
Median	346.00	168.00	11.00	1
Mode	346	168	10	1
Standard deviation	60.397	35.046	3952	0.203
Variance	3645.628	1228.224	15.621	0.041
Minimun	125	58	4	1 “Negative”
Maximun	545	232	36	2 “Positive”

**Table 2:** Levels of LDH in Adolescent Athletes from Sports Centers in Tacna—2023.

LDH			
Level	Normal Values	*n*	%
Low	0–200 U/L	8	2.5%
Regular	200–400 U/L	298	91.4%
High	400 U/L and above	20	6.1%

**Table 3:** Levels of Total CK in Adolescent Athletes from Sports Centers in Tacna—2023.

TOTAL—CK			
Level	Normal Values	*n*	%
Low	0–24 U/L	0	0.0%
Regular	24–195 U/L	244	74.8%
High	195 U/L and above	82	25.2%

**Table 4:** CK-MB Levels in Adolescents from Sports Centers in Tacna—2023.

CK-MB			
Level	Normal Values	*n*	%
Regular	0–25 U/L	323	99.1%
High	24 and above	3	0.1%

**Table 5:** CK-MB/CK Ratio in Adolescent Athletes from Sports Centers in Tacna—2023.

Relative CK-MB/CK Ratio		
Level	*n*	%
CKMB/CK < 4%	23	7.1%
4% < CKMB/CK < 25%	303	92.9%
CKMB/CK > 25%	0	0.0%

**Table 6:** Levels of C-Reactive Protein in Adolescent Athletes from Sports Centers in Tacna—2023.

C—Reactive Protein		
Level	*n*	%
Negative	312	95.7%
Positive	14	4.3%

**Table 7:** Frequency of Muscular Alterations in Adolescents from Sports Centers in Tacna—2023.

Frequency of Alterations		
Level	*n*	%
1 Marker	99	88.4%
2 Markers	11	9.8%
3 Markers	2	1.8%
4 Markers	0	0.0%
Total	122	100%

**Table 8:** Values of key muscle damage markers in individuals with elevated levels.

ID	CK (U/L)	LDH (U/L)	CRP (U/L)	CK-MB (U/L)
1	210	420	1.2	20
2	230	430	1.5	22
3	250	410	1.1	24
4	270	450	1.8	23
5	190	390	1.3	19
6	260	440	1.4	21

**Table 9:** Correlation matrix of muscle damage markers.

	CK (U/L)	LDH (U/L)	CRP (U/L)	CK-MB (U/L)
CK (U/L)	1000	0.260	0.237	−0.143
LDH (U/L)	0.260	1000	0.483	0.780
CRP (U/L)	0.237	0.483	1000	0.194
CK-MB (U/L)	−0.143	0.780	0.194	1000

## Data Availability

All data generated or analyzed during this study are included in this article. Further inquiries can be directed to the corresponding author.
